# Journeying together: spousal experiences with prostate cancer in Ghana

**DOI:** 10.3332/ecancer.2024.1692

**Published:** 2024-04-11

**Authors:** Evans Osei Appiah, Ezekiel Oti-Boadi, Ninon P Amertil, Rosina Afotey, Honest Lavoe, Isabella Garti, Awube Menlah, Eric Kwesi Ntiako Sekyi

**Affiliations:** 1 Nursing Department, Purdue University, 425 South River Road, West Lafayette, IN, USA; 2 Department of Nursing, Heritage Christian College, PO Box AN16798, Amasaman, Accra, Ghana; 3 Nursing Department, School of Nursing and Midwifery, Valley View University, Oyibi, Ghana; 4 Valley View University, Oyibi, Ghana; 5 Accra-Psychiatric Hospital, Accra, Ghana; 6 University of Charles Darwin, Brinkin, Northern Territory Australia; a https://orcid.org/0000-0002-6730-4725; b https://orcid.org/0000-0002-0683-1572

**Keywords:** spousal experience, prostate cancer, emotional well-being, physical well-being

## Abstract

**Background:**

Prostate cancer (PCa) is a significant global health concern for men. In Sub-Saharan Africa, PCa rates witnessed a 69% increase from 1990 to 2010. Despite this, there is a dearth of literature examining the experiences of spouses of men with PCa in Africa, as the majority of studies concentrate primarily on men.

**Methods:**

The study used a qualitative exploratory design, conducting in-depth face-to-face interviews with a semi-structured guide. Participants were selected through purposive sampling, with 35 recruited. Data was recorded, transcribed verbatim, and analysed using content analysis, resulting in 2 themes and 11 subthemes.

**Results:**

The research revealed that spouses providing care for husbands with PCa faced notable effects on their physical and emotional well-being. Notably, they reported experiencing leg pains due to prolonged sitting by their partners, as well as disruptions in sleep and a loss of appetite triggered by the hospital smell.

**Conclusion:**

Women encounter challenges in caring for their partners with PCa. Understanding these experiences will contribute to improving public support and assistance. Future studies should concentrate on developing interventions to help them cope with these challenges.

## Introduction

Cancer poses a growing global public health challenge, with GLOBOCAN 2020 reporting 19.3 million new cases and nearly 10 million cancer-related deaths in 2020 [1, 2]. In Sub-Saharan Africa, prostate cancer (PCa) rates witnessed a 69% increase from 1990 to 2010 [3]. Recently, in 2020, there were 1,414,259 new cases of PCa reported worldwide, resulting in 375,304 related deaths [4]. Notably, the incidence of PCa is particularly elevated among Black men of West African ancestry in the Caribbean Islands, the United Kingdom, and West Africa [5]. Additionally, the overall pooled incidence of PCa in Africa was reported at 21.95 per 100,000 population, with a median incidence of 19.47 per 100,000 population [3].

In Ghana, studies on PCa predominantly focus on medical treatments like surgery, radiotherapy, hormone therapy, and chemotherapy, alongside non-medical options such as herbal remedies [6]. However, limited literature exists on the experiences of women with spouses diagnosed with PCa. Moreover, patients with cancer in Sub-Saharan Africa and Ghana, including PCa, often delay seeking treatment until the disease has advanced and metastasized to vital organs [7, 8].

Several studies have been done on PCa, but most of these studies focus on the disease progression, effects on the patient and the partner’s early detection and treatment, and quality of life after surgical treatment [9–11]. However, there is a lack of literature on the experiences of spouses of men with PCa in Ghana. The purpose of this study was to explore this aspect. The purpose of this study was to explore the experiences of spouses of men with PCa in Ghana.

## Methodology

### Research design

For the purpose of this study, a qualitative exploratory design was employed to fully understand the participants in terms of experiences of spouses of men with PCa. This design does not seek to provide the final solutions to the research questions but simply explores the research topic with some level of depth [12].

### Research setting

The study was conducted at Korle-Bu Teaching Hospital, the premier healthcare facility in Accra and renowned as one of the best in West Africa. Established in 1923, it currently has a bed capacity of 2,000, making it the largest teaching hospital in Ghana and the second largest in West Africa. The hospital accommodates approximately 40, 75 and 88 patients in the children’s, male, and female wards, respectively. The facility is known for receiving major referrals nationwide, leading to the establishment of the oncology center, The National Centre of Radiotherapy and Nuclear Medicine, in 1997. This collaborative effort between the Government of Ghana, the Ministry of Health, the International Atomic Energy Agency, and the Ghana Atomic Energy Commission treats various cancers such as cervical, throat, and breast cancers. Additionally, the hospital’s genitourinary department specialises in minimally invasive surgeries like percutaneous nephrolithotomy for kidney stone removal and provides treatment for disorders like penile blockage and urinary tract infections. The majority of interviews were conducted on weekends at participants’ homes, with only a few taking place at the facility.

### Sampling technique

A purposive sampling technique was used to select the participants of the study. This is the sampling technique where the researchers use their discretion in selecting members to participate in the study [13]. This technique was deliberately employed so as to gather the needed data specifically for the research. Only participants who met the inclusion criteria were involved in the study.

### Sample size

The sample size was determined by the goal of gathering comprehensive information until saturation. Researchers interviewed participants until no new information emerged, concluding the study when saturation was reached after the 35th person.

### Inclusion criteria

Participants included in the study were only spouses of patients with PCa at Korle-Bu Teaching Hospital who were willing to partake in the study.

### Exclusion criteria

Certain criteria disqualify potential participants from the study. Specifically, the researchers excluded spouses of patients who were mentally unsound or ill, those whose partners did not have PCa, those whose partners were not receiving treatment for PCa at Korle Bu Teaching Hospital (KBTH), and participants who did not provide consent.

### Data collection procedure

Ethical clearance was secured from the Dodowa Health Research Centre Institutional Review Board. An introductory letter, along with the clearance letter, was submitted to the administration of KBTH. Face-to-face interviews were conducted in English at the facility, scheduled at convenient dates and times for the respondents. The study involved a total of 35 participants.

Field notes were meticulously recorded when deemed necessary. Respondents were prompted to elaborate on their perspectives. With respondents’ consent, the face–face indebt interviews were audio-recorded using a voice recorder. The data was guided by semi-structured interviews designed by the authors based on literature. The researchers diligently documented all field encounters and non-verbal cues in her notebook. Interviews typically lasted between 45 minutes to 1 hour. The semi-structured interview guide included several questions such as:

Can you tell me about yourself? Can you describe your partner’s condition? How has your husband’s diagnosis affected you physically? How has your husband’s diagnosis affected you emotionally?

How have you been coping with the situation?

### Data analysis techniques

**Figure 1.**
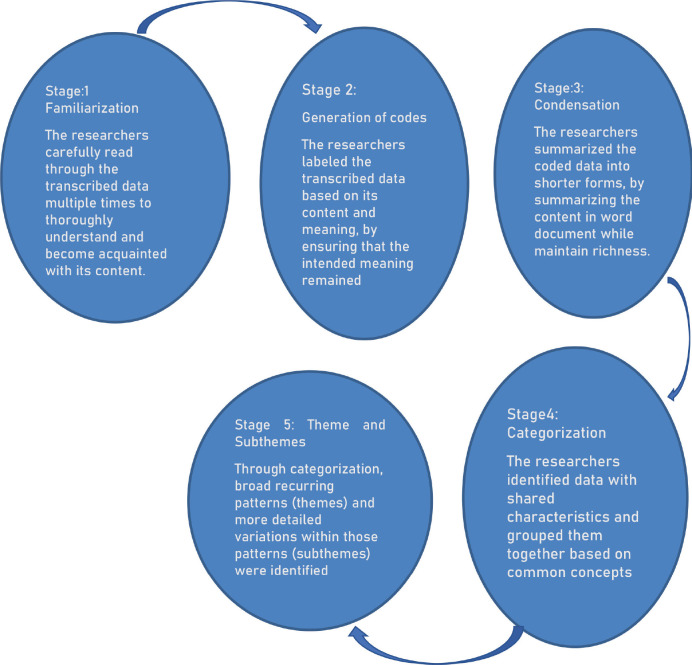
Stages of the content analyses.

This study used inductive content analysis for data analysis. Hence data was condensed by shortening the text by preserving its meaning, coded, categorised by grouping together codes related to each other and interpreted into themes and sub-themes. Data was analysed manually in a word document by the first four authors. The analyses of data were done based on five stages identified in the literature (Figure 1) [14].

### Methodological rigor

#### Credibility

The responses of these participants were verified at the end of each interview session before concluding the data collection. Each interview was transcribed verbatim and coded before subsequent interviews were conducted to assist the researcher to relate with the responses and content. Also, the findings of the study were studied by the research supervisors.

#### Transferability

Transferability was ensured by the researcher by making sure that, a detailed description of the methodology and procedures guiding interviews were provided.

#### Dependability

To ensure dependability in this study, the researcher implemented the research methodology strictly throughout the study. The study setting, steps, and procedures carried out were described clearly. The researcher also kept an audit trail.

#### Confirmability

To ensure confirmability, the researcher reflected on his views and predisposition to make sure that they do not influence the findings of the study. Audio recordings were transcribed verbatim and direct quotes used to support emerging themes. Furthermore, an audit trail comprising field notes, notes from member checks, and summaries were used to provide information on the context and background of an interview to enhance analysis.

## Results

### Analysis of socio-demographic data

The sociodemographic data of the study revealed that 35 participants were included. Among them, the majority (18) fell within the age range of 35–40 years (51.42%), while the minority (7) were aged 45–50 years (20.00%), representing the least recorded age group in the study. Regarding educational attainment, the majority of participants held a Basic Education Certificate Examination (BECE) qualification (15, 42.85%), followed by West African Senior Secondary Certificate Examination (WASSCE) holders (9, 25.71%). Additionally, participants with diplomas were represented by six individuals (17.14%), while those holding degrees comprised the minority (5, 14.28%). See Table 1 for further details

#### Organisation of the themes

There were two main themes and 11 sub-themes as presented in Table 2.

### The physical well-being of spouses of men with PCa

The interviews of the participants revealed five distinct sub-themes, which are as follows: Sleeping disturbances, Legs pains from prolonged sitting from spouses, Insufficient time for physical activities, loss of appetite due to hospital smell, and feeling of tiredness from providing assistive care to the husband.

### Sleep disturbances caused by husbands’ condition and environmental changes

*Participants in this study shared various occasions when they struggled to sleep, citing factors such as environmental changes and concerns about their husband’s illness. The ensuing are few of the statements made by the participants:*

*‘Ever since my husband’s admission, I’ve been struggling to fall asleep. Despite my desire to rest, s\][leep eludes me whenever I lie down. I consulted the doctor about my problem, and he suggested that my emotional state is causing the sleeplessness. However, he reassured me that everything will turn out alright.’* **p12**

*‘Regarding my sleep pattern after my husband’s admission to Korle-Bu, it has been very poor. I struggle to sleep for more than half an hour due to the unfamiliar surroundings. My sleeping habits have been disrupted, and I am hopeful that I will be able to sleep soundly again soon*.’ **p2**

*‘It’s difficult for me to sleep when the breadwinner of our family is unwell. I’ve realised that my husband’s sickness is the reason for my insomnia. I have managed to get a few hours of sleep, but since his condition is critical, I need to monitor him constantly. In case of any complications, I need to be alert and report immediately’* **p10**

Even though most participants in this study voiced out that they cannot sleep, a handful of the participants also echoed that they do not have issues with their sleeping patterns. Below are some narrations from the analysis;

*‘I’m able to sleep at night because my husband, who is now 80 years old, has accomplished a lot in his life. After struggling for 6 years, he seems exhausted, and if death were to come calling, we would welcome it without hesitation, I am not worried so much like the initial stages of the condition.’* **p17**

### Leg pain resulting from extended periods of sitting

Participants also reported experiencing leg pains from prolonged sitting. They mentioned that they often had to spend extended periods sitting by their husbands to provide care, which led to discomfort and pain in their legs. The following are some of the participants’ accounts:

‘*Before my husband’s diagnosis, I wasn’t used to sitting for prolonged periods, but now I find myself in a similar position. He has been hospitalised for the last 5 months, and as the only family member caring for him, I have to be with him constantly. My legs have been aching, but there are no beds available for us here. I could sleep on the bench outside, but I am unwilling to leave his side’* **p23**

*‘Ever since my husband was admitted three months ago, I have been experiencing pain in my legs. I am unable to lie down and have to remain seated all the time. Although I have been taking Diclofenac to alleviate the pain, it seems to be worsening and becoming chronic.’* p**17**

‘*I know very well that this is a hospital but at least the hospital should try and provide us with better chairs to sit on. This chair isn’t good and that is the same chair I have been sitting on until my legs pains started.’* p**9**

### Insufficient time for family caregiver’s physical activities

Based on the findings of this study, many participants voiced their struggle to engage in adequate physical activity due to their responsibilities towards their spouse, leading to a shortage of time. Below are some of the participants’ remarks:

‘*Well, due to my husband’s condition, I have not been able to visit the gym. Here am I, I can’t remember the last time I visited the gym as I used to do with my husband. But I know he will soon be back on his feet and we will both exercise again as we used to exercise.’* p**3**

‘*Hmmmmm, today marks exactly 3 weeks I have not exercised because my sport’s man (husband) is sick. As soon as my husband recovers, we will start with our jogging again. For now, I cannot leave my husband and go for jogging*.’ p**11**

‘*I know exercise is good and have been walking for 1 hour every day to keep myself active but these few years I do not even think about been active since my husband is my priority.*’ p**19**

### The decrease in appetite caused by the odor present in the hospital setting

Study participants noted a decreased appetite characterised by a lack of hunger or inclination to eat, attributing it significantly to the hospital odour. Here are some participant remarks regarding the appetite loss linked to the hospital smell:

‘*As you may agree, the hospital environment is not pleasant, with the smell of urine and medication filling the air. Consequently, I often lose my appetite for food when I am here. I have only managed to eat a biscuit and drink some water when I sat outside on the bench.’* p**25**

‘*How do you expect me to eat in such environment? My husband is really trying. I have not been able to eat unless I am out of the ward. I lost appetite since the very first day I enter this ward*. *But I joined my husband yesterday during lunch and have to manage to eat because I did not want him to feel bad.’ p****13***

Even though most participants in this study stated that they had lost appetite due to the smell in the hospital, a handful of the participants had no challenge eating in such environments. Below are some comments from the analysis:

‘*I believe that those who complain about the smell and loss of appetite may simply not be hungry. In my case, both my husband and I have been able to eat without any issues. However, I am more concerned about my husband’s condition than my own appetite.’* p**16**

### Feeling of tiredness from providing assistive care to husband

The study documented that prolonged stay in the ward led to feelings of exhaustion among some participants, attributed to the care they provided for their husbands. Their sentiments were captured as follows:

‘*I must admit that I have lost a lot of strength lately. Despite being 50 years old, I have been assisting with my husband’s daily activities, such as turning him over. It has taken a toll on me, and I am exhausted, but I cannot complain as my priority is caring for my husband.’* p**11**

‘*When?? When exactly will my husband be discharged home so that I can rest. Tiredness from providing assistive care to my husband is what I face every day and his condition is not getting better. I feel as if I am even sick. I wish I can get a break but who will take care of him?’* p**15**

Although the majority of the participants in this study expressed feelings of tiredness from providing assistive care to the husband, a handful of the participants were motivated to render care at all times without feeling tired.

‘*I am highly motivated and do not believe that I will become tired from caring for my husband. I feel that he would have done the same for me if our roles were reversed, and that thought keeps me going. My momentum to provide care and assistance for him remains strong.’* p**4**

#### The psycho-social well-being of spouses of men with prostate cancer

Psychosocial well-being is a broad concept that encompasses emotional and psychological well-being, as well as social well-being. During the study, participants shared their perspectives on psychosocial well-being, and six sub-themes emerged from their interviews. These sub-themes were: Fear of losing their partners, Spouses’ opinion about the diagnosis, Experience during hospitalisation of their partners, Fear of partners becoming infertile, Fear of decreased sexual performance, and Ineffective coping.

### Fear of losing their partners

The primary concern expressed by women in this study revolved around the fear of losing their partners, leading to significant worry due to the deep emotional bond they shared. They conveyed apprehension about their ability to cope without their husbands in the event of their passing due to cancer diagnosis. Below are excerpts of comments made by some participants;

‘*How will you feel if you find out that your husband has PCa? Honestly I don’t want to lose my husband because I love him so much and living without my husband will make like very difficult for me.’* p**7**

‘*Awwww, this is easy oooh, I have been told that my husband has less than a year to live, what borders me is that he can die at any moment leaving me alone on this world to suffer I always share tears when I remember the good times we shared together*.’ p**18**

Even though there is fear of losing their partners, participants have echoed that they prayed and believe that God will intervene.

‘*Well, even though my sweet husband has already been diagnosed with PCa, as for me I don’t believe it ooooh, I know very well that with God all things are possible, and my God will heal my husband. God is the physician who can do all things*.’ p**15**

### Spouses’ opinion about the diagnosis

Participants provided insights into their understanding of PCa. Many expressed a lack of comprehensive knowledge about the disease. Here are some of the comments shared by the participants:

‘*Hmmmmm, me! I don’t know anything about PCa ooo, I have never heard it before. Even before we came here to Korle-Bu my husband told me he wasn’t feeling well, just for me to get here and be told he has PCa. As at now, I can’t say anything about PCa itself because I do not know how this condition came about and why he should develop PCa?’* p**15**

‘*Seriously speaking I don’t know anything about the PCa the doctors and nurses are talking about. When we got admitted here, I thought it was malaria, and after going for several lab investigations and it saying my husband is having cancer. The Cancer alone is making me scared (crying….). I don’t know where to start from.*’ **P6**

Some participants expressed confidence that their husbands’ illnesses could be effectively managed by healthcare professionals.

‘*Well, even though PCa is dangerous as a disease, I know it can be managed, so that is why I brought my husband to the hospital, and I know everything will be fine*.’ **P4**

### Experience during hospitalisation of their partners

Participants in this study conveyed their negative experiences, including the financial strain of treatments and anxiety about medical procedures like catheterisation. The following quotes offer an in-depth analysis of the results pertaining to this sub-theme:

‘*Hmmmm, just for the past 1 month that we have been here at the ward, it hasn’t been easy with me at all. we have spent almost 10,000 Ghana Cedis Just on diagnostic investigations alone, we are yet to begin cancer drugs and I learnt it cost about 5, 000 Ghana cedis.’* P**12**

‘*The diagnosis has been challenging to my husband and I. My husband was not able to urinate so a rubber has been inserted in his penis which he has been complaining that it is painful and another problem is that with this catheter I am afraid we cannot have sex again.’* P**8**

Even though some participants expressed their fear due to the catheter *insi-tu*, and the amount of money spent, a handful experiences the effects of the side effects of the drugs**.** ‘*Well, all I can say it that it hasn’t been easy for the past 2 months we have been on admission. My husband all of a sudden is having problems with thinking and remembering things. But I was told by a nurse it is the side effects of the medication that he is on that is causing all this*.’ P**17**

### Fear of partners becoming infertile

Additional conversations with participants revealed their worries about the potential infertility of their partners as a result of PCa. The following paragraphs delve into a thorough examination of these findings.

‘*Hmmmm, what is scary now is that my husband can become infertile due to the disease but I don’t for it to happen. Before we got married, we agreed on getting four kids and just 5 years of marriage with two male kids he is been diagnosed with PCa this means that if he becomes infertile, we will not have any female child.’* P**11**

‘*I know the prostate forms an active part in reproduction so if it is cancerous then I am afraid that reproduction will be affected and I am 40 years now almost closed to menopause and we had just one child so I am just hoping it does not affect his fertility’* P**10**

Other participants mentioned that they would prefer their husbands to be alive and be infertile than dying due to the condition.

*‘Even if my husband becomes infertile today and remains alive, I will be grateful unto the Lord. By the grace of God, we have been blessed with five kids already and that this the number of children we asked God to bless us with. At this point, my husband’s life matters to me than him being infertile.’ P* ***9***

### Fear of decrease sexual performance

Participants in the study voiced apprehension regarding the possibility of diminished sexual performance due to the condition. The subsequent paragraphs offer an in-depth analysis of these findings.

‘*My husband and I have been married for almost 16 years now and I know what my husband can do when it comes to sex. My husband is good. My fear that the PCa might affect his sexual performance and at 42 years I am still active and enjoy sex.’* P14

‘*My husband is not a 1-minute man even at the age of 50 years, he can really perform and that is one of the reasons why I love him so much. He can satisfy me like four times in a week and I even heard frequent sex helps to prevent this cancer so I don’t know why he should be diagnosed at this age.’* p**21**

Even though some participants in this study voiced the fear of their partner decrease sexual performance, few participants on the other hand expressed that their husband will remain the love of their life.

‘*Even if my husband sexual performance reduce, he will still remain the love of my life because he is a loving husband and after all I am 50, I do not even enjoy sex as compared to when I was young.’* p7

### Ineffective coping

Through data analysis, it was found that partners of individuals with PCa were adopting ineffective coping strategies. Some participants expressed difficulty in adjusting to their partners’ new condition due to the challenges they were facing. The following are comments made by a few participants:

‘*Hmmm, I don’t know what is happening to my husband ooooh. This is our second months on admission and to be honest my husband communication pattern has changed. I am not getting why all of a sudden*. My husband now speaks slowly like a baby, which is something new I have never noticed my husband doing.’ p**20**

‘*Hmmmm, my dear it is not easy oooh, my husband is now and then complaining about tiredness meanwhile is always sitting or lying-in bed. Just after walking around the ward for sometimes, he will complain of tiredness, and I have observed based on his facial expression that he is depressed.’* p**6**

Some participants also attested to the fact that they cope effectively with all their daily activities due to the support they receive from family members and church.

‘*This is not to say that I am not bordered oooh, but the truth is that family members are supporting me to the extent that I go about my daily activities without any issue. It was difficulty at the beginning but now my brothers & sisters’ in-laws are really supporting us*.’ p**18**

## Discussion of findings

The participants attributed their insomnia to emotional distress caused by their spouse’s condition, impacting their ability to fall asleep and concentrate during the day. Prioritising caregivers’ emotional well-being is essential for quality care. Psychological support can enhance their quality of life and contribute to better care. Another study found anxiety and depression in women with husbands diagnosed with prostatic cancer, while 115 participants in a different study revealed higher emotional distress and difficulty sleeping in spouses of prostatic cancer patients [15, 16].

In the study, most participants reported leg pain due to prolonged sitting while providing constant support to their husbands. This underscores the physical strain of caregiving, particularly for those required to stay in one position for extended periods. A study also found that prolonged sitting leads to leg discomfort [16].

The study found that many participants faced challenges in exercising due to spending extensive time at the hospital with their husbands diagnosed with prostatic cancer. This struggle emphasizes the need for support and resources, such as hospital gyms, to help caregivers maintain their physical and mental well-being. In a related study, it was suggested that physical activity interventions for older adults with cancer and their family caregivers should incorporate strategies promoting self-efficacy, realistic goals, motivation and social support [17].

Participants in this study observed that the hospital environment’s odour adversely affects their appetite, resulting in challenges with eating and subsequent weight loss. When spouses struggle to consume adequate nutrition, they may lack the energy needed to provide care for their husbands. This finding underscores the importance of prioritising improvements to the cleanliness and ambiance of the hospital environment to mitigate these challenges. Similarly, a separate study found that cancer patients also faced eating difficulties due to unpleasant hospital doors [18].

The present study unveiled that approximately two-thirds of participants harboured concerns about losing a spouse to PCa, expressing apprehension about the prospect of sole caregiving for children. This underscores the profound emotional toll on families, particularly women, necessitating comprehensive support measures. A parallel study corroborated these findings, indicating a shared fear among spouses of PCa patients and underscoring the widespread emotional repercussions [19]. Consequently, policy interventions should prioritise the implementation of robust support mechanisms, including psychological assistance tailored to caregivers’ needs.

The study found that a significant number of participants were unaware of their husbands’ PCa diagnosis until the hospital visit, indicating a lack of prior knowledge about the condition. This underscores the need for enhanced education and awareness campaigns on PCa, as many individuals may lack access to information about the condition and its risks. A study discovered a similar lack of knowledge among patients diagnosed with PCa, emphasising the need for education [20].

The study revealed that approximately two-thirds of participants expressed worries about potentially losing a spouse to PCa, particularly concerning the prospect of sole caregiving for children. This underscores the significant emotional strain faced by families, especially women, indicating the urgent need for comprehensive support measures. These findings align with a parallel study, which also highlighted a shared fear among spouses of PCa patients, underscoring the widespread emotional impact across affected families [21, 22]. To address the financial burdens associated with PCa treatment, proactive government interventions, such as subsidising treatment costs through insurance coverage or allocating funds from non-governmental agencies, are essential. Additionally, non-governmental organisations could play a crucial role in assisting families by providing support for both medical and non-medical expenses, helping alleviate financial pressures and ensuring families can navigate the costs associated with treatment effectively.

The study participants feared potential impotence in their spouses due to PCa, causing distress, especially for those with family planning aspirations. The emotional impact on family planning highlights the necessity for comprehensive support strategies addressing the physical and emotional needs of those affected by the disease. Similarly, a systematic review of the literature affirmed that some participants had a fear of developing erectile dysfunction following PCa surgery [23].

The study revealed that many participants were concerned about their husbands experiencing a decline in sexual performance due to PCa. They emphasised that despite their husbands’ age, they remain sexually active and fear the potential impact of the disease on their sexual function. A similar finding noted that while sex plays a crucial role in marriage, decreased sexual performance and fear of impotence was one of the major fears spouses exhibited [24, 25].

## Limitations and strengths

The study is constrained by its qualitative design, which restricted data collection to a small number of participants. Additionally, the study’s focus on spouses of PCa patients at one facility limiting the generalisability of the results. However, the study’s strength lies in its ability to deeply explore and elicit rich first-hand information and experiences related to PCa. Future research could consider expanding to include family dyads to capture more nuanced data, as well as conducting experimental studies to develop interventions aimed at supporting these families.

## Conclusion

In conclusion, it is evident that women face significant challenges when caring for partners with PCa. Enhancing our understanding of these experiences is crucial for improving public support and assistance. Future research endeavours should prioritise the development of interventions aimed at helping caregivers cope with these challenges effectively. Recognising and addressing the needs of caregivers in the context of PCa care is essential. By gaining insight into the unique challenges faced by spouses, healthcare providers and policymakers can devise more effective strategies to support and assist them in their caregiving role.

## Conflicts of interest

There are no conflicts of interest related to the publication of this work.

## Funding

The study was not funded.

## Consent for publication

Not applicable.

## Ethical considerations

The researcher obtained an ethical clearance from the Dodowa Health Research Centre Institutional Review Board (DHRCIRB/190/09/22. A purposive sampling permits the recruitment of participants who met the inclusion and exclusion criteria and were willing to partake in the study after an informed consent form was obtained from them.

## Figures and Tables

**Table 1.** Socio-demographic data of the respondents.

Variables	Frequency (*n* = 35)	Percentages (%)
Age group		
25–30	10	28.57
35–40	18	51.42
45–50	7	20.00
Educational status		
BECE	15	42.85
WASSCE	9	25.71
Diploma	6	17.14
Bachelor’s degree	5	14.28
Religion		
Christians	22	62.85
Muslims	9	25.71
Traditionalists	4	11.42
Marital status		
Married	24	68.57
Co-habitation	8	22.85
Divorce	3	8.57
Occupation		
Trader	18	51.42
Nurse	10	28.57
Teacher	6	17.14
Cleaner	1	2.85
Ethnicity		
Akan	21	60
Ewes	4	11.43
Ga-Dangme	6	17.14
Others	4	11.43

**Table 2.** Summary of themes and their respective sub-theme.

Themes	Subthemes
**The physical well-being of spouses of men with PCa.**	Sleep disturbances caused by husbands’ condition and environmental changes. Leg pain resulting from extended periods of sitting. Insufficient time for physical activities Loss of appetite due to hospital smell Feeling of tiredness from providing assistive care to husband
**The psycho-social well-being of spouses of men with PCa**	Fear of losing their partners Spouses’ opinion about the diagnosis Experience during hospitalisation of their partners Fear of partners becoming infertile Fear of decrease sexual performance Ineffective coping
